# Human moral decision-making through the lens of Parkinson’s disease

**DOI:** 10.1038/s41531-021-00167-w

**Published:** 2021-03-02

**Authors:** Giorgia Ponsi, Marina Scattolin, Riccardo Villa, Salvatore Maria Aglioti

**Affiliations:** 1 Department of Psychology Sapienza University of Rome and CLNS@SAPIENZA Roma, Istituto Italiano di Tecnologia, Genova, Italy; 2 IRCCS Fondazione Santa Lucia, Roma, Italy

**Keywords:** Human behaviour, Parkinson's disease

## Abstract

Parkinson’s disease (PD) is a neurodegenerative disorder characterized by the loss of dopaminergic neurons in the basal ganglia (BG) and thalamocortical circuitry. While defective motor control has long been considered the defining symptom of PD, mounting evidence indicates that the BG are fundamentally important for a multitude of cognitive, emotional, and motivational processes in addition to motor function. Here, we review alterations in moral decision-making in people with PD, specifically in the context of deceptive behavior. We report that PD patients exhibit two opposite behavioral patterns: hyper- and hypo-honesty. The hyper-honest subgroup engages in deception less often than matched controls, even when lying is associated with a monetary payoff. This behavioral pattern seems to be linked to dopaminergic hypo-activity, implying enhanced harm avoidance, risk aversion, non-impulsivity, and reduced reward sensitivity. On the contrary, the hypo-honest subgroup—often characterized by the additional diagnosis of impulse control disorders (ICDs) and dopamine dysregulation syndrome (DDS)—deceives more often than both PD patients without ICDs/DDS and controls. This behavioral pattern appears to be associated with dopaminergic hyperactivity, which underpins enhanced novelty-seeking, risk-proneness, impulsivity, and reward sensitivity. We posit that these two complementary behavioral patterns might be related to dysfunction of the dopaminergic reward system, leading to reduced or enhanced motivation to deceive. Only a few studies have directly investigated moral decision-making in PD and other neurodegenerative disorders affecting the BG, and further research on the causal role of subcortical structures in shaping moral behavior is needed.

## Introduction

Parkinson’s disease (PD) is a progressive, multisystem neurodegenerative disorder that affects 2–3% of the population over 65 years of age^1^. PD is characterized by a premature loss of dopaminergic neurons in the substantia nigra pars compacta (SNpc) and widespread intracellular protein (α-synuclein) accumulation called Lewy pathology^2^.

The substantia nigra is part of the basal ganglia (BG), a group of subcortical nuclei that also comprises the caudate nucleus, the putamen, the globus pallidus, and the subthalamic nucleus (STN)^3^. The caudate nucleus and the putamen form the dorsal striatum (DS), the largest component of the BG. The ventral striatum (VS) is composed of the nucleus accumbens (NAcc), the olfactory tubercle, and the most ventral sections of the caudate and the putamen. Crucially, dopaminergic neurons in the NAcc and the ventral tegmental area (VTA) form the core of the reward circuit^4^. The dorsal and ventral sections of the striatum are implicated in different processes: the DS is mainly involved in sensorimotor and cognitive control, while the VS supports reward and motivation. The BG can be considered as a kind of motivation-to-movement interface that allows interaction between reward circuits supporting motivated behavior and brain regions involved in cognition and motor control^5^.

Loss of dopaminergic neurons in PD occurs predominantly in the nigrostriatal dopamine pathway (which connects the SNpc with the DS), but it also affects the mesocortical dopamine pathway (which connects the ventral tegmentum and medial SNpc with frontal areas), leading to dopamine depletion within the frontal cortex^6^. Interestingly, the progressive nature of the clinical symptoms observed in PD seems to be explained by the spatiotemporal progression of dopamine depletion within the striatum and its cortical afferents^7^.

The primary motor symptoms of PD, include bradykinesia (slowness of movement), hypo-akinesia (reduced frequency of spontaneous movements), resting tremor (involuntary rhythmic movement of a body part), and rigidity (increased muscle tone producing stiffness of the limb)^8^. In addition to motor control disorders, PD is also characterized by non-motor symptoms: neurocognitive impairments^9^, psychiatric disorders^10^, olfactory deficits^11^, and autonomic^12^ dysfunctions.

PD has traditionally been classified as a purely motor disorder and the BG were considered to be exclusively devoted to sensorimotor coordination, and the selection, initiation, and execution of movement. However, experimental evidence indicates that motor and non-motor circuits of the BG are spatially segregated^13^ and that output projections of the BG target multiple cortical sectors that are not directly involved in sensory or motor function, including the prefrontal cortex (PFC)^14^. Moreover, research has shown that the BG play a nearly ubiquitous role in cognition^15^, motivation^16^, and emotion^17^. A recent meta-analysis based on 5809 human imaging studies has provided a “psychological map” of striatal function, identifying five distinct zones^18^: (1) VS (stimulus value and related stimulus-driven motivational states), (2) anterior caudate (action-outcome value and incentive behavior), (3) posterior putamen (sensorimotor processes, including their affective qualities; e.g., pain and pleasure), (4) anterior putamen (social and language-related functions; e.g., social value, empathy), and (5) posterior caudate (executive functions and cognitive control). This new evidence explains how striatal damage—as seen in PD or Huntington’s disease (a monogenic neurological disorder characterized by prominent cell loss and atrophy in the caudate and putamen^19^)—can produce a wide range of cognitive, motivational, affective, and social symptoms, even before the deterioration of motor performance. Given the role of nigrostriatal dopaminergic loss in the emergence of parkinsonian motor and non-motor symptoms, PD constitutes a clinical model for understanding the multi-faceted impact of (i) structural damage of BG/thalamocortical circuitry, and (ii) dopaminergic medication on higher-level psychological functions, particularly moral cognition/behavior.

In this review, we will first summarize recent findings on motivational and socio-emotional dysfunction in PD. Then, we will describe the existing experimental evidence describing alterations in moral cognition in PD patients. We argue that deficits in motivated behavior arising from degeneration of the BG and side effects of the dopaminergic medication may provoke two opposite patterns of moral behavior: hyper- and hypo-honesty.

## Motivational and socio-emotional alterations in Parkinson’s disease

In addition to the well-known motor impairments that characterize PD, recent data suggest that dopamine-related deficits in motivated behavior could be ubiquitous in PD patients, regardless of medical status (i.e., on vs. off medication) and apathy^20^. Clinical observations and experimental reports have long suggested the existence of a typical premorbid parkinsonian personality^21^, characterized by introversive, conscientious, industrious, non-impulsive, risk-averse, inflexible, law-abiding, and rigidly moral personality traits. Notably, PD patients exhibit reduced novelty seeking^22^ and higher tendencies for harm avoidance^23^ compared to controls, as confirmed by a recent meta-analysis^24^. Changes in personality and motivational tendencies often precede the clinical motor symptoms of PD and are thought to be partially mediated by dopaminergic striatal deficits^25^ and are therefore known as hypo-dopaminergic behavioral patterns. Accordingly, dopamine agonist administration in PD patients performing a feedback-based probabilistic classification task increases novelty seeking and reward processing, while decreasing punishment processing^26^. In addition, harm avoidance personality scores are positively correlated with ^18^F-dopa uptake in the caudate nucleus^27^. Novelty seeking is also negatively correlated with dopamine D_2_ receptor availability in the insular cortex^28^. Non-addictive personality traits and behaviors might be linked to defective reward processing^29^ (as evidenced by a reduced reward positivity^30^—RewP—a positive-going event-related potential occurring at frontocentral electrode sites ~300 ms after the presentation of rewards vs. non-rewards) and reward-related learning^31^. In line with this view, a recent study showed that PD patients suffering from dopamine depletion exhibit selective impairments in reward learning and that dopaminergic medication can reestablish the ability to maximize reward^32^.

Conversely, an almost opposite behavioral pattern, characterized by hedonism, enhanced novelty-seeking, impulsivity, and repetitive compulsive behaviors^33^ (e.g., pathological gambling, hypersexuality, compulsive shopping, binge eating, hobbyism, and punding—complex prolonged, purposeless, and stereotyped motor behavior), has been described in PD patients diagnosed with impulse control disorders (ICDs) and dopamine dysregulation syndrome (DDS), an addiction-like state characterized by compulsive medication use. These disorders share an increased involvement of the dopaminergic system, leading to similar hyper-dopaminergic behaviors. In fact, DDS is primarily associated with higher potency dopamine replacement therapy, such as levodopa^34^. Similarly, impulsive–compulsive traits typical of PD ICD+ (Parkinson’s disease patients with a diagnosis of ICDs) are also related to hyperactivation in subcortical and cortical brain structures associated with reward processing and inhibitory control^35^. These impulsive-compulsive traits may be related to alterations in the reward circuit: PD patients with pathological gambling disorder (PG-PD) exhibit increased dopamine release in the VS in response to winning compared to PD patients without pathological gambling (non-PG-PD)^36^, indicating that winning money may have a higher motivational value for PG-PD than non-PG-PD patients.

Together with alterations in motivation and personality, socio-emotional functioning (e.g., empathy and facial emotion recognition) also seems to be impaired in PD^37^. In particular, both cognitive and affective components of the Theory of Mind (ToM, the ability to make inferences about others’ mental states—including thoughts, intentions, and emotions—in social contexts) are affected in PD^38^. According to a recent meta-analysis^39^, PD patients have significantly impaired emotion recognition compared to controls—an effect found across modalities (visual, auditory) and task types. The same pattern emerges in Huntington’s disease^40^, suggesting that poor motor control caused by striatal neurodegeneration may dampen patients’ facial muscle contractions in response to perceived emotional expressions, leading to defective emotion recognition.

Other accounts suggest that PD patients show a reduced capacity to make script-based inferences, which may affect their social behavior and intuition^41^. However, only a few studies directly investigated the domain of social cognition and social decision-making in PD. Some suggest that dopaminergic medication may (i) decrease sensitivity to emotion bias in decisions involving faces^42^ and (ii) increase altruistic punishment behavior in PD ICD+^43^, a result that might be explained by enhanced sensitivity to negative feedback and increased impulsivity. Other studies using the Trust Game (an economic game that measures trusting behavior) showed that, in comparison to controls, PD patients are less trusting of others^44^ and tend to trust avatar faces more than human faces^45^. In addition, PD patients with STN deep brain stimulation (STN-DBS) implants overrate their own performance relative to other participants’, indicating self-overestimation and increased willingness to compete^46^. Consistent with this, a recent study showed that the capacity for strategic learning in the presence of competitive opponents seems to be preserved in patients with BG damage^47^. Dysfunctional social behavior may also be linked to alterations in social reward processing: this might be the case for PD ICD + patients who show reckless generosity^48^, e.g., compulsions to give away money and gifts.

Taken together, evidence suggests that PD may affect functions implicated in social interaction, although it is not clear whether this condition can also bias the decision-making process toward honesty or dishonesty. To examine this issue in more detail, the next section focuses on dysfunction in moral cognition.

## Altered moral cognition in Parkinson’s disease

Morality refers to a system of norms, values, and customs adopted by a specific cultural group to guide behavior and sociomoral conduct^49^. Moral judgment and moral decision-making are prominent phenomena investigated within the realm of moral cognition.

### Moral judgment

Moral judgment refers to the process of judging events or behaviors that have a moral component. It is typically investigated using experimental paradigms in which participants must judge morally ambiguous situations (moral dilemmas). Only a few studies to date have investigated moral judgment in PD.

Fumagalli et al.^50^ investigated whether STN local field potential was modulated during a moral task in PD patients bilaterally implanted with STN electrodes for DBS. The moral task consisted of morally conflictual, morally non-conflictual, and neutral statements. Patients were asked to read each sentence and report whether or not they agreed with it. Results showed that reaction times (RTs) were significantly longer when processing moral conflictual vs. moral non-conflictual sentences. Also, low-frequency (5–13 Hz) STN activity increased significantly more during morally conflictual vs. morally non-conflictual or neutral sentences. As the authors themselves acknowledged, these results imply that low-frequency STN oscillations are involved in conflict processing, but they are not specifically devoted to processing moral content. The same group investigated whether STN-DBS can alter moral judgment in PD patients with bilateral STN-DBS implants compared to a control group of PD patients without implants^51^. STN stimulation had no effect on RTs or moral judgments, and this was independent of stimulation state (on vs. off) and the presence of DBS implants.

Rosen et al.^52^ presented PD patients with 20 everyday moral dilemmas (10 minimally emotional and 10 highly emotional in nature) and asked participants whether or not they would behave in altruistic or egoistic ways in those particular circumstances. The results showed that healthy controls employ ToM to make strategic egoistic decisions in minimally emotional dilemmas, while PD patients’ decisions are not based on ToM. In another study, Rosen et al.^53^ presented PD patients with the same dilemmas and found that PD patients made significantly more egoistic decisions than healthy controls in highly emotional moral dilemmas. According to the authors, this behavioral pattern may be related to dysfunction in the frontostriatal circuits that support the integration of executive functions, ToM, and empathy in the resolution of moral conflict. This is in line with findings on patients with ventromedial PFC (VMPFC) lesions, who show utilitarian moral judgment when presented with highly emotional^54^ and personal^55^ moral dilemmas. However, moral dilemma paradigms of this kind have been criticized because they are unlikely to reflect how moral decision-making naturally occurs in daily life^56^.

### Moral decision-making

Moral decision-making refers to the ability to choose an optimal course of action - with respect to moral norms and values - among multiple alternatives, usually with direct or indirect consequences for oneself and others^57^ (e.g., helping or harming, telling the truth or lying, sacrificing a personal benefit to help, or prevent the suffering of another individual). Unlike moral dilemmas, where people make hypothetical judgments, moral decision-making paradigms require participants to actively make multiple decisions that are good proxies for decisions that occur in reality.

A specific type of moral decision-making is deceptive decision-making. Deception is a specific social behavior in which one individual (the deceiver) deliberately attempts to persuade another to accept something as true even though they know it to be false^58,59^. Complex social manipulations like tactical deception and the generation of “dishonest signals” are present in human and non-human primates, indicating notable sophistication of deception processes^60^. Interestingly, the frequency of deceptive behavior used for social manipulation can be predicted by neocortex volume in 18 primate species^61^, suggesting that the evolutionary expansion of this brain structure might have been driven by the increased cognitive demands of navigating increasingly complex social arenas.

Deciding whether to lie or not requires both ToM and counterfactual thinking^62^, two functions that are impaired in PD patients. Saltzman et al.^63^ reported that PD patients were impaired in the Spy Model Task, a paradigm that involves planning a course of action to deceive another person. McNamara et al.^64^ found that PD patients are impaired in generating counterfactuals (i.e., mental representations of alternative events with respect to reality) and that this deficit may be related to PFC dysfunction.

Previous studies investigating deceptive decision-making in PD reported mixed results. Most found evidence of a “hyper-honest” behavioral pattern in people with PD or essential tremor^65–67^. Abe et al.^65^, for example, found that PD patients’ ability to lie was impaired in an instructed deception task in which PD patients and healthy controls were asked to either tell the truth or lie to another individual about their familiarity with previously seen visual stimuli. Critically, resting-state ^18^F-fluorodeoxyglucose Positron Emission Tomography (PET) revealed that this impairment was significantly correlated with hypometabolism in the PFC (left dorsolateral and right anterior prefrontal regions) in the PD patient group and could not be explained in terms of the severity of cognitive deficits.

Similarly, using the guilty knowledge task, Mameli et al.^66^ explicitly asked patients with PD or essential tremor to either tell the truth or lie about their familiarity with a visual stimulus presented on the screen that was (or was not) previously chosen by them. This study confirmed that, compared to healthy controls, PD and essential tremor patients were impaired in producing deceptive responses, suggesting that this impairment may represent a common cognitive feature of movement disorders characterized by fronto-subcortical circuit dysfunctions.

Abe et al.^67^ also employed an incentivized prediction task in which PD patients were given the opportunity for dishonest gain by spontaneously lying about the accuracy of their predictions relative to the location (left or right side) of a visual stimulus. In line with the studies reviewed above, PD patients’ percentage of self-reported wins was lower than controls’, confirming the hypothesis that PD patients show reduced cheating behavior even when confronted with the opportunity to obtain financial benefits.

Interestingly, studies suggest that a subgroup of PD patients—namely those diagnosed with pathological gambling—may exhibit a tendency for dishonesty. Brusa and colleagues^68^ found that PG-PD patients had an enhanced tendency to lie compared to non-PG-PD patients: PG-PD patients’ scores were significantly higher in all three validity subscales—lying, lying frequency, and defensive behavior—of the Minnesota Multiphasic Personality Inventory 2 (MMPI-2) questionnaire. Interestingly, even PG-PD patients whose score was in the non-pathological range showed higher values in the lying frequency subscale compared to the non-PG-PD group. Moreover, a single-case clinical report^69^ showed that a patient diagnosed with multiple system atrophy with predominant parkinsonism (MSA-P) and a premorbid history of addiction exhibited very high scores in the Lie scale (particularly the egoistic lie sub-scale) of the Big Five Questionnaire-2 and tried to voluntarily hide pathological behaviors.

## Dysfunctional deceptive decision-making in PD: a joint contribution of cortical and subcortical brain alterations

The aforementioned studies investigating deceptive decision-making in PD reported mixed evidence: some found a hyper-honest behavioral pattern^65–67^ in PD patients, while others reported the polar opposite, hypo-honesty^68,69^. These discrepancies may be related to methodological differences: studies employing active deceptive decision-making tasks found that PD and essential tremor patients behave more honestly than healthy controls^65–67^ even when they can benefit from lying (i.e., dishonest gain), whereas studies investigating personality aspects linked to deception found that PD patients engage in cheating more often than controls^68,69^.

Two different arguments can explain deceptive behavior abnormalities in PD: one cognitive/executive and one motivational.

Support for the former comes from studies suggesting that cognitive and executive impairments related to PFC dysfunctions are possible causes of the reduced propensity for deception. For example, Abe et al.^65^ hypothesized that the hyper-honest behavioral pattern found in PD is due to cognitive deficits resulting from pathological changes in PFC. Indeed,^18^F-fluorodeoxyglucose PET revealed that reduced deceptive behavior significantly correlated with decreased metabolic rate in the left dorsolateral and right anterior PFC of PD patients. In line with this finding, recent meta-analyses investigating the neurocognitive basis of deception found increased activation in the frontal lobes for deceptive vs. truthful responses^70,71^, suggesting a pivotal role of executive control processes in deceptive behavior. Moreover, the dorsal ACC, the right temporoparietal junction/angular gyrus, and the bilateral temporal pole are consistently more active in social-interactive vs. noninteractive deception tasks^71^.

Support for the latter comes from studies suggesting that a defective dopaminergic reward system may represent the substrate for reduced motivation to deceive. Abe et al.^67^ argued that a dysfunctional reward system is causally relevant for a decreased propensity for deception, despite the possibility of dishonest gain. This is consistent with previous neuroimaging studies showing greater activation of subcortical structures like the amygdala^72^, caudate nucleus^73–77^, NAcc^78^, and thalamic nuclei^79^ in deceptive vs. truthful responses.

To summarize, alterations in deceptive decision-making found in PD may be the result of both cortical and subcortical brain dysfunction due to neurodegeneration within the BG/thalamocortical circuitry. This view implies that deceptive behavior is sustained by cognitive processes such as perspective-taking, ToM, moral reasoning, and conflict processing. In addition, subcortical brain structures implicated in reward processing, emotional processing/regulation, and social interaction may play a role in the modulation of deceptive behaviors.

**Fig. 1:**
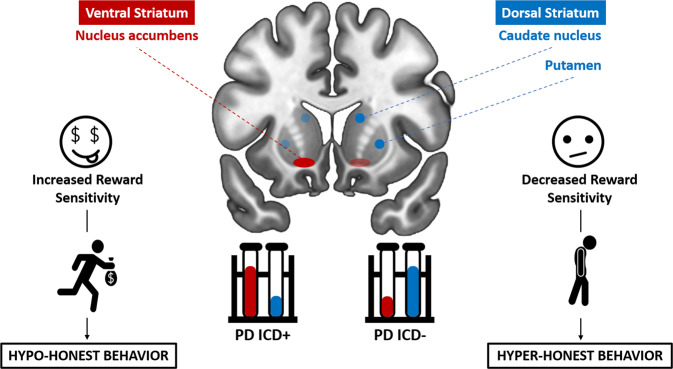
Alterations of dopaminergic striatal levels may lead to hypo- or hyper-honesty in PD. Schematic overview of the role of dopaminergic striatal levels in explaining the behavioral transition from the purported dynamic balance of health condition to the unbalance that leads to hypo- or hyper-honesty in PD. The dopaminergic imbalance between the ventral and dorsal striatum may alter the brain motivation system and thus enhance/decrease the salience of reward-related stimuli. Altered incentive salience (i.e., “wanting”) may in turn explain the hypo- and hyper-honest behavioral tendencies expressed by PD patients during deceptive decision-making. This model is inspired by a recent meta-analysis^90^. The hypo- and hyper-honest behavioral tendencies have been displayed respectively on the left and right side of the figure only for representational purposes. No hypothesis on brain lateralization has been proposed.

Here, we propose that a defective brain motivation/reward system may explain both the hyper- and hypo-honest behavioral tendencies expressed by PD patients during deceptive decision-making (see Fig. 1).

## Can the unbalance of striatal dopamine levels explain hypo- and hyper-honest behavioral tendencies in PD?

As discussed above, PD patients classically show hypo-dopaminergic behaviors (e.g., absence of impulsivity, risk aversion, and lower reward sensitivity) and a personality profile characterized by reduced novelty seeking and enhanced harm avoidance. There is a close relationship between morality and harm avoidance: aversion to harm is an influential force in human morality^80^. Indeed, motor resonance to observed immoral actions is suppressed more in high harm-avoiders compared to low harm-avoiders^81^, and serotonin was found to promote moral behavior by enhancing harm aversion for oneself^82^ and for others^83^. Consequently, enhanced harm avoidance in PD patients may explain their reduced willingness to cheat and behave dishonestly.

The picture is completely reversed for PD patients who developed ICDs and DDS. In this case, the personality profile seems opposite to that of typical PD patients, featuring high impulsivity, addictive behaviors, risk-seeking, and reward-seeking. This motivational shift could explain why this subgroup of PD patients have a greater tendency to lie. There is a crucial link between enhanced reward processing and dishonest behavior: heightened neural responses to anticipated reward in the NAcc predict the frequency of dishonest behavior^84^, enhanced reward-related neural activity is associated with a greater likelihood of deception^85^, and dispositional greed predicts unethical decisions in an incentivized corruption game^86^. Thus, the hypo-honest behavioral pattern found in PD ICD+ may be due to enhanced salience of reward-associated stimuli.

In particular, the opposing behavioral patterns found in PD patients with and without ICDs may be related to differential neurodegeneration of striatal sections between the two subpopulations, specifically a greater dysregulation in VS-mediated processes in PD ICD+. Indeed, current theoretical models^87,88^ suggest that dopaminergic input to the DS provides a reinforcing signal that binds the representation of stimuli and responses into stable associations (stimulus-response learning), whereas the dopaminergic input to the VS plays a key role in rewarding and affective aspects of motivated behavior (reward prediction and stimulus-reward learning). Furthermore, the DS is mainly involved in associative and motor aspects of decision-making, while the VS supports value-encoding^89^.

Crucially, a recent meta-analysis^90^ on the PET/SPECT (Single-Photon Emission Computed Tomography) dopaminergic striatal correlates of ICD in PD showed that compared to PD ICD- (Parkinson’s disease patients without a diagnosis of ICDs), PD ICD+ patients have (i) lower dopaminergic transporter levels in the DS (indicating more severe nigrostriatal dopaminergic loss), and (ii) increased presynaptic dopamine release in the VS in response to reward-related tasks. According to these findings, the dopaminergic imbalance between DS and VS in PD ICD+ may constitute a possible neural substrate for ICD in PD. In line with this dopaminergic imbalance hypothesis, PG-PD patients show greater dopamine release in the VS when winning compared to controls^36^, and PD ICD+ patients exhibit hyperactivation in brain structures associated with reward processing in comparison with PD ICD− patients and controls^35^.

Further, PD patients with single and multiple ICDs show increased VS dopamine release in response to reward cues compared to PD ICD- patients, even when the cues are unrelated to the specific ICD (e.g., PG-PD patients confronted with food-related cues)^91,92^. The incentive-sensitization theory of addiction^93^ states that repeated exposure to psychostimulant drugs induces long-lasting neuroadaptations in the VS reward circuitry, which becomes hypersensitive to drugs and drug-associated cues. A psychological function of this neural system is the attribution of incentive salience or “wanting”—a process that imbues stimuli with “magnet-like” qualities and elicits an approach toward them. Thus, the pharmacological induction of ICD in vulnerable PD individuals may resemble the process of global incentive-sensitization to reward, with enhanced ventral striatal dopamine neurotransmission resulting in excessive “wanting” motivation that also spills over to non-drug rewards (e.g., money).

Together, these results suggest that it is plausible that striatal dopaminergic imbalance responsible for enhanced incentive salience to reward in PD ICD+ patients may play a central role in the development of their dishonest behavioral tendencies. The relationship between striatal dopaminergic imbalance and dishonesty is likely indirect and mediated by incentive salience. Indeed, dishonest or cheating behavior often represents a means to obtain rewards or to avoid punishments in daily life.

## Conclusions and future perspectives

Brain damage or dysfunction induced by neurological diseases can profoundly alter different higher-order human functions including moral^94,95^, religious^96^, and criminal^97^ behavior. Here, we reviewed the evidence for altered deceptive decision-making in PD, an issue of fundamental theoretical and clinical importance that has attracted comparatively little attention thus far (see^98^ for a previous review). We found that two opposite behavioral patterns linked to distinct motivational tendencies can be observed in PD patients: hyper- and hypo-honesty. Unfortunately, only a few studies have directly investigated deceptive decision-making in PD and other neurodegenerative disorders affecting subcortical structures. These studies present several methodological limitations, namely: (i) the employed behavioral paradigms were not ecologically valid: participants were told when to lie or not to lie, so no spontaneous decision was made during the tasks; (ii) the employed behavioral paradigms were not “social”: contrary to what happens in reality, lies and truths were not directed toward another social agent; (iii) the employed decision tasks did not imply real (or presumed real) consequences on other social agents (a factor that is likely to influence participants’ decisions); (iv) the medical status of the patients (on vs. off dopaminergic medication) was often mixed; and (v) the presence of ICDs was not always assessed.

Cognitive and executive functions (particularly cognitive control^78^) supported by the PFC play a pivotal role in human deceptive behavior: correlation analyses carried out in the reviewed articles^65–67^ suggest that there is a relationship between dysfunctional cognitive/executive function and decreased dishonesty. However, further research is needed to clarify the exact cognitive mechanisms (e.g., failure in inhibiting true responses and producing deceptive ones)—and their possible interaction with the motivational deficits previously described—underlying impaired deception ability in PD patients.

Future studies should thoroughly investigate the relationship between motivation, reward, and moral behavior in patient populations characterized by subcortical damage. For example, DBS and recording of electrophysiological activity in specific subcortical nuclei offer a unique opportunity to study the involvement of subcortical structures in different aspects of moral cognition. Existing DBS evidence^50,51^ has explored the phenomenon of moral judgment. However, the impact of DBS on moral decision-making (and on moral behavior in general) has yet to be investigated. Future PD research should also aim to distinguish the role of structural damage within the BG (especially in distinct sections like DS and VS) from the pharmacological effects of dopaminergic medications. Finally, in order to investigate spontaneous deceptive decisions that involve real consequences for oneself and others, we believe that more ecologically valid experimental paradigms (e.g., the Temptation to Lie Card Game^99–102^, the Coin-flip/Coin Tossing Task^84^, or the Spot the Difference Task^78^) should be employed. These future steps may ultimately lead to a better understanding of the role of the BG and other subcortical structures in shaping social and moral behavior in PD as well as in healthy individuals.

## Data Availability

Data sharing not applicable to this article as no datasets were generated or analyzed during the current study.
